# Changing Family Dynamics Through Childhood: Exploring Household Chaos as a Moderator of Bidirectional Effects Between Parent and Child Behaviors

**DOI:** 10.1016/j.jaacop.2025.08.001

**Published:** 2025-08-12

**Authors:** Bonamy R. Oliver, Jon Heron, Jasmine A.L. Raw, Jane Gilmour, Emily Midouhas

**Affiliations:** a University College London, London, United Kingdom; b University of Bristol, Bristol, United Kingdom

**Keywords:** parenting, problem behavior, externalizing problems, household chaos, longitudinal studies

## Abstract

**Objective:**

Harsh parenting and childhood externalizing behaviors can form bidirectional, reinforcing dynamics that set the stage for adverse outcomes. In community samples, little is known about contextual factors that moderate this bidirectionality; we examined household chaos as a key candidate.

**Method:**

Using the United Kingdom’s Millennium Cohort Study (MCS; 17,115 families), we tested moderation by chaos of pathways between main-carer reports of harsh parenting and externalizing behaviors over child ages 3, 5, and 7 years.

**Results:**

Findings supported the mutual nature of parent and child behaviors through childhood. Despite 2-year intervals, there was also evidence for interactions between chaos and harsh parenting in predicting later externalizing problems, and between externalizing problems and chaos in predicting later harsh parenting. As hypothesized, perceptions of high chaos in the home exacerbated associations between harsh parenting and later externalizing problems. Unexpectedly, children’s externalizing problems had a weaker influence on harsh parenting in the context of higher chaos (or, indeed, a stronger influence in low-chaos homes). Acknowledging our anticipated small effect sizes, several interpretations are discussed. For example, parents perceiving higher chaos may filter out the excessive stimulation of their children’s externalizing problems and be less reactive to them, or lower home chaos may reflect a need for calm and control in parents who are particularly reactive to their children’s externalizing behaviors.

**Conclusion:**

Negative, reinforcing parent–child dynamics are seen outside clinical contexts over time. Exploring moderators of this bidirectionality may offer a nuanced understanding of family processes that hold the key to prevention and intervention.

## Plain language summary

Parents and children influence each other’s behavior over time. Understanding how the family home environment strengthens or weakens these patterns may inform ways to mitigate long-term conflictual cycles in the family. Analyzing the UK Millennium Cohort Study (MCS, 17,115 families), this study found that household chaos (eg, disorganization, lack of routine, and sense of rush rather than calm) may change the mutual association between parental harshness and child externalizing behavior over time. The results suggest complex dynamics between parent and child behavior, particularly in the context of household chaos.

The importance of parents for children’s social, emotional, and behavioral adjustment has long been acknowledged.^1^ Since Baumrind’s influential parenting styles,^2^ broad constructs of positive parenting (eg, warmth, sensitivity, responsiveness) and negative parenting (eg, criticism, punitive discipline) have been shown to function largely as protective and risk factors respectively for children’s development. These patterns are seen across cultures, despite some differences in specific parenting approaches and their prevalence.^3^ Nevertheless, Bell’s assertion^4^ that associations between parenting and children’s outcomes should be conceptualized as “child-on-parent” effects in concert with “parent-on-child” effects remains fundamental. Indeed, influential models^5^, ^6^, ^7^ have embraced the notion of parent–child bidirectionality: children’s behavior influences parents’ behavior, just as parents influence their children. A substantial body of empirical work in this area has a keen focus on bidirectional associations between harsh parenting (eg, verbal or physical hostility, coercion) and children’s externalizing behaviors (eg, aggression, oppositionality).^8^, ^9^, ^10^ Because continuation of these negative dynamics in the parent–child system is understood to be likely to have long-term consequences,^6^ interrupting them is a core feature of many intervention programs.

Understanding factors that change bidirectional parent–child processes—for better or worse—outside of the clinical context is crucial, offering information likely to lead to a better understanding of prevention as well as intervention effectiveness. Children with persistent externalizing problems below thresholds for clinical intervention are at risk for adverse outcomes,^11^ and the amelioration of such difficulties may reduce clinical and societal burden in the long term, as well as improving health equity.^12^^,^^13^ Although little is known about moderation of bidirectional parent–child processes in community samples, factors previously considered include temperament,^14^ self-regulation,^15^ and attachment,^16^ as well as contextual factors such as economic hardship^17^ and cultural normativeness.^18^ Here, we turn our attention to the potential role of household chaos.^19^

Household chaos references a cluster of characteristics of the home environment such as disorganization, a lack of routine, and a sense of rush rather than calm.^20^ Although chaos associates with sociodemographic factors such as poverty and overcrowding, it is far from a substitute,^20^ and is posited to have independent adverse effects on both adults’ and children’s emotional and behavioral regulation.^21^ This may manifest for children as, for example, emotion dysregulation and externalizing problems, and for parents as a reduced capacity for attentive and nurturing parenting.^22^ Importantly, multiple differences at individual and population levels (eg, expectations, norms) can differentiate contexts and their influence on development. Moreover, there is growing recognition that children are contingently influenced by the context in which they develop, moving away from assumptions about environments that constitute “risk” such that individual differences in outcomes reflect responsiveness to context, with adaptive as well as maladaptive responses apparent.^23^ In line with this, we do not view household chaos as an objective measure of “risk” with external notions of what is appropriate or otherwise, but rather as a subjective perception of the home environment by reporter. Parent perceptions of chaos vary considerably in community samples and associate with behaviors of both parents and children in the home.^22^^,^^24^ As such, we suggest that chaos is a strong candidate for the moderation of mutual dynamics between parents and children.

Household chaos has received considerable attention in relation to harsh parenting and children’s externalizing problems. High levels of chaos have been shown to amplify associations between these constructs, with chaos considered to exacerbate the deleterious effects of parental harshness on children’s behavior.^24^ Importantly, considering the bidirectional model, it is also plausible that perceived chaos exacerbates the impact of children’s behavior problems on parents’ capacity for staying calm and positive with their children. We know of just one published study that has examined this notion.^15^ In a sample of 311 children, the authors examined bidirectionality of harsh parenting and child externalizing problems over late childhood to early adolescence (ages 9, 11, and 14 years) with moderation by household chaos reported once (age 14 years). Using mother, father, and child reports, the authors found moderation by parent—not child—perceptions of chaos, for the pathway from externalizing problems at age 9 years to harsh parenting at age 11. However, the direction of results was unexpected, with these child-on-parent effects apparent only at low or mean levels of parent-reported chaos at age 14. The authors concluded that their findings may indicate perceptions of chaos to subsume associations between child externalizing problems and later parenting, or that parents in more regulated households are more attuned to their children’s externalizing behaviors and respond with increased harsh parenting. Nevertheless, in our study of younger children, we expected high chaos to amplify the child-on-parent pathways. This was based on research showing stronger links between externalizing problems and harsh parenting in families as a function of socioeconomic hardship, attributed to parental stress and fewer social support resources commonly being available.^25^ Although not a proxy for socioeconomic disadvantage, we hypothesized that chaos would have similar effects because of its relationship with stress and the notion that, even if social support is available, parents perceiving high levels of chaos may be unable to access it.^26^

In the current study, we explored moderation by chaos of bidirectional parent–child processes from early to mid childhood, because the transition from preschool to school years (age 5 years in the United Kingdom) introduces new demands on children’s behavior, readjusts family relationships, and necessitates changes to family routines.^27^ In addition, although physical punishment is more prevalent in preschool years than in later childhood,^28^ other harsh parenting strategies driven by negative parental emotional responses persist in their relevance.^29^ Given the role of chaos in disrupting proximal family processes^19^ and its influence on parent and child emotional and behavioral regulation,^22^ we expected that parent–child dynamics during this period would be susceptible to the influence of chaos.

### Current Study

In the first study of its kind, we capitalized on the United Kingdom’s large longitudinal cohort study, the Millennium Cohort Study (MCS), to explore moderation by household chaos of bidirectional parent–child processes over the preschool to early school years (child age 3, 5 and 7 years). The statistical power offered by MCS affords the detection of interactive effects that are likely to be small, given the intervals (2 years) between timepoints. We accounted for construct stability and all main effects in our longitudinal path analysis to explore moderation of paths from parenting to later child behavior and child behavior to later parenting. Specifically, we examined the following: chaos at age 3 years as a moderator of the path between harsh parenting at age 3 and externalizing behavior at age 5; chaos at age 5 years as a moderator of the path between harsh parenting at age 5 and externalizing behavior at age 7; chaos at age 3 years as a moderator of the path between externalizing behavior at age 3 and harsh parenting at age 5; and chaos at age 5 years as a moderator of the path between externalizing behavior at age 5 and harsh parenting at age 7. We hypothesized that chaos would exacerbate the deleterious influences of harsh parenting on later externalizing problems and of child externalizing problems on later harsh parenting.

## Method

### Sample and Procedure

MCS recruited from all UK births within a year from September 1, 2000. The sample is disproportionately stratified to ensure adequate participant numbers from: the 4 countries in the United Kingdom (England, Wales, Scotland, Northern Ireland), electoral wards with sociodemographic disadvantage (those whose value on the Child Poverty Index in 1998 to 1999 was above 38.4%,) and, in England, higher proportions of people of color (more than 30% Black or Asian in the ward at 1991 census). This represents the top 25% cut-off threshold of disadvantaged wards in England and Wales, and a slightly greater fraction in Scotland and Northern Ireland (full details in Hansen *et al.* and Plewis^30^^,^^31^). Ethical approval for the MCS was obtained from NHS Multi-Centre Ethics Committees, with parents giving explicit written informed consent before data collection. We used data for one child per family (first-born of twins or triplets) from data collection sweeps 1 to 4 (child ages 9 months, 3 years, 5 years, and 7 years). Main carers (97% biological mothers) were interviewed at home, reporting child externalizing problems and harsh parenting at sweeps 2, 3, and 4, and household chaos at sweeps 2 and 3. Confounders were collected at sweeps 1 and 2 (ethnicity, child sex recorded at birth) to include the new families joining MCS at sweep 2, except for poverty status, which was collected at sweep 2. Our analytic sample comprized families with data on our key variables (child externalizing problems, harsh parenting, household chaos) for at least one sweep (N = 17,115; 88.9% of MCS families).

### Measures

#### Harsh Parenting

Main-carer reports on 3 items from the Conflict Tactics Scales^32^ measured the use of physical (smacking) and verbal (shouting, telling off) discipline when the child misbehaved. Items were rated on a 5-point Likert frequency scale from never (coded 1) to daily (5), and summed such that higher scores indicated more frequent use of these negative parenting tactics (maximum score = 15; α = 0.67, 0.67, and 0.67 at sweeps 2, 3, and 4, respectively).

#### Child Externalizing Problems

Main-carer reports from the Strengths and Difficulties Questionnaire (SDQ)^33^ measured children’s externalizing problems, using the conduct and hyperactivity-inattention behaviors subscales. Each subscale has 5 items, rated not true (coded 0) to certainly true (2). Sample items include “often has temper tantrums or hot tempers” and “often fights with other children or bullies them” (conduct problems subscale), and “restless, overactive, cannot stay still for long” and “easily distracted, concentration wanders” (hyperactivity-inattention problems subscale). Subscales were combined into an overall externalizing problems score (α = 0.78, 0.78, and 0.80 at sweeps 2, 3, and 4, respectively) as recommended for population samples.^34^

#### Household Chaos

Three items from the Confusion, Order and Hubbub Scale (CHAOS)^35^ were reported by main carer: namely, “the atmosphere in my home is calm,” “I can’t hear myself think in my home,” and “it is really disorganized in our home.” Items were rated on a 5-point Likert scale from 1 (strongly agree) to 5 (strongly disagree). We reversed and summed items to create a total score whereby a high score indicated more perceived household chaos (α = 0.68 and 0.67 at sweeps 2 and 3, respectively).

#### Confounders

Confounders (Table 1) were binary (dummy-coded) indicators of main-carer education (university degree or higher by age 7 years), poverty status (below poverty line set for equivalized net family income at 60% of UK national median), child female sex assigned at birth, and child ethnicity (Black, Indian, Mixed, Other, Pakistani/Bangladeshi, White).

**Table 1:** Descriptive Statistics and Correlations Among Study Variables

Sociodemographic variables (confounders)		%							
Ethnicity									
Black		3.67							
Indian		2.58							
Mixed		3.04							
Other		1.44							
Pakistani/Bangladeshi		6.86							
White		82.40							
Parental education (university degree or higher)		19.25							
Below the poverty line		33.95							
Child sex at birth (female participants)		48.92							
Two-parent family		79.91							
		Mean (SD*)*							
No. of people at home		4.20 (1.31)							
No. of siblings at home		1.21 (1.09)							
			**Correlations**						
**Study variables**		**Mean (SD*)***	**1.**	**2.**	**3.**	**4.**	**5.**	**6.**	**7.**
Age 3 y									
1.	Child externalizing problems	6.75 (3.83)	—						
2.	Harsh parenting	9.33 (2.41)	0.34	—					
3.	Household chaos	7.01 (2.19)	0.31	0.18	—				
Age 5 y									
4.	Child externalizing problems	4.84 (3.44)	0.61	0.24	0.24	—			
5.	Harsh parenting	8.35 (1.97)	0.25	0.56	0.14	0.33	—		
6.	Household chaos	7.28 (2.28)	0.26	0.15	0.47	0.32	0.22	—	
Age 7 y									
7.	Child externalizing problems	4.77 (3.61)	0.54	0.22	0.22	0.71	0.27	0.27	—
8.	Harsh parenting	8.11 (1.91)	0.25	0.51	0.12	0.29	0.62	0.18	0.36

Note: Below the poverty line: set for equivalized net family income at 60% of the UK national median household income. Descriptive statistics and correlations are given for uncentered variables. For all correlations (n = 10,074-15,446), *p* < .001.

### Data Analysis

**Figure 1:**
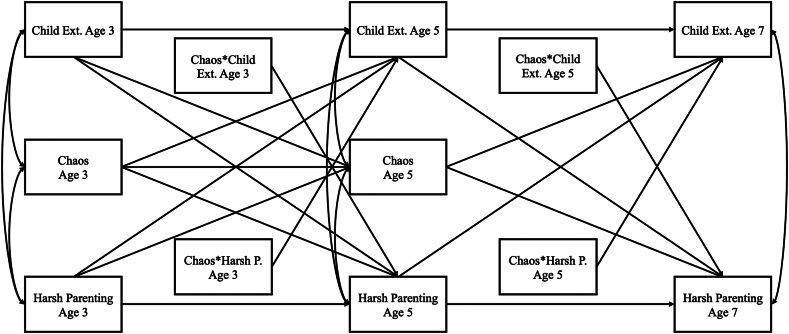
Path Model ***Note:****All paths adjusted for confounding variables. Within-time correlations at ages 5 and 7 years are between residuals, but are illustrated here as between constructs for clarity because of the complexity of the model. Child Ext. = child externalizing problems; Harsh P. = harsh parenting.*

Analyses were pre-planned but not pre-registered. Data preparation and preliminary analyses were conducted in Stata v.18. Our structural equation model (Figure 1) was estimated using full information maximum likelihood (FIML) in MPlus v.8, with a maximum likelihood estimator with robust standard errors (MLR). Main variables were centered. For each path, we adjusted for dummy-coded confounders as well as the 9 MCS area-strata dummies to account for sampling design, as follows: England–advantaged, England–ethnic, England–disadvantaged (reference group), Wales–advantaged, Wales–disadvantaged, Scotland–advantaged, Scotland–disadvantaged, Northern Ireland–advantaged, and Northern Ireland–disadvantaged.^30^ Missingness is summarized in Table S1, available online.

We derived associations between harsh parenting and externalizing problems within and across time, adding stability and main effects of chaos at ages 3 and 5 years. We also included interaction terms (chaos at 3 × harsh parenting at 3; chaos at 5 × harsh parenting at 5; chaos at 3 × externalizing problems at 3; chaos at 5 × externalizing problems at 5) to test moderation by household chaos for the cross-construct cross-time paths only, according to our hypotheses. To aid interpretation of significant interactions, we estimated marginal effects with average chaos (mean) and high/low chaos (±1 SD respectively), plotting at each chaos level predicted values of harsh parenting by child externalizing behavior and vice versa, as relevant. Predicted values were plotted for the reference groups of dummy variables. Plots were within data bounds, but cannot and should not be used to determine the size of effects.^36^

## Results

Descriptive statistics and correlations among variables are given in Table 1.

Correlations between all variables were in expected directions and ranged from small to moderate in magnitude. As is to be expected, the highest correlations were those indicating within-domain stability over time and, for harsh parenting and externalizing problems, when measured closer in time (ie, age 3-5 years; age 5-7 years). All correlations across domains were small to moderate and in expected directions (*r* = 0.12-0.36). Our structural equation model (Figure 1) examined longitudinal associations between variables, included moderation by chaos of hypothesized paths, and accounted for confounders. Our model fit was satisfactory, based on the following: standardized root mean squared residual (SRMR) <0.08^37^; confirmatory factor analysis (CFI) ≥0.95, root mean square error of approximation (RMSEA)<0.07 and Tucker–Lewis index (TLI) ≥0.95,^38^ although the complexity of the model to conservatively allow the inclusion of all confounders rendered TLI a little low (SRMSR = 0.015; RMSEA = 0.057; CFI = 0.959; TLI = 0.779).

Model findings (Table 2; Table S2, available online) reflected simple correlations: that is, there was considerable stability for all domains, and expected within-time associations between constructs. Accounting for these pathways, small significant associations remained between harsh parenting and externalizing problems over time (age 3-5 yers and ages 5-7 years). Notably, implying bidirectionality, the magnitude of cross-construct paths across ages was similar for higher levels of harsh parenting in association with higher levels of externalizing problems 2 years later, and for higher levels of externalizing problems in association with later higher levels of harsh parenting. We found chaos to be playing its expected predictive role at ages 3 and 5 years on harsh parenting and externalizing problems 2 years later (at 5 and 7 years, respectively). Albeit not a primary focus, higher levels of harsh parenting and externalizing problems at age 3 years were associated with more chaos at age 5.

**Table 2:** Path Model Results: Associations Between Externalizing Problems and Harsh Parenting Over Time Moderated by Household Chaos

	Standardized Estimate	SE	*p*
Predicting externalizing problems at 5 y			
Externalizing problems at 3 y	0.55	0.01	<.001
Harsh parenting at 3 y	0.03	0.01	<.001
Household chaos at 3 y	0.05	0.01	<.001
Harsh parenting at 3 y × household chaos at 3 y	0.02	0.01	.005
Predicting harsh parenting at 5 y			
Harsh parenting at 3 y	0.54	0.01	<.001
Externalizing problems at 3 y	0.07	0.01	<.001
Household chaos at 3 y	0.03	0.01	.002
Externalizing problems × household chaos at 3 y	−0.01	0.00	.290
Predicting chaos at 5 y			
Household chaos at 3 y	0.41	0.01	<.001
Harsh parenting at 3 y	0.04	0.01	<.001
Externalizing problems at 3 y	0.10	0.01	<.001
Predicting externalizing problems at 7 y			
Externalizing problems at 5 y	0.66	0.01	<.001
Harsh parenting at 5 y	0.05	0.01	<.001
Household chaos at 5 y	0.03	0.01	<.001
Harsh parenting at 5 y × household chaos at 5 y	0.02	0.01	.009
Predicting harsh parenting at 7 y			
Harsh parenting at 5 y	0.59	0.01	<.001
Externalizing problems at 5 y	0.10	0.01	<.001
Household chaos at 5 y	0.03	0.01	.001
Externalizing problems at 5 y × household chaos at 5 y	−0.02	0.01	.037

Note: For more information on path model results, see Figure 1. All variables were centred and all pathways accounted for confounding variables and area strata. SE = standard error.

Three of our 4 hypothesized interactions suggested weak significant moderation by household chaos of associations between harsh parenting and externalizing problems (the fourth hypothesized pathway was of similar magnitude). Specifically, chaos and harsh parenting at age 3 were found to interact (chaos at 3 × harsh parenting at 3) in the prediction of children’s externalizing problems at age 5 (b = 0.02, SE = 0.01, *p =* .005), chaos and harsh parenting at age 5 were found to interact (chaos at 5 × harsh parenting at 5) in the prediction of externalizing problems at age 7 (b = 0.02, SE = 0.01, *p =* .009), and chaos and children’s externalizing problems at age 5 were found to interact (chaos at 5 × externalizing problems at 5) in the predication of harsh parenting at age 7 (b = −0.02, SE = 0.01, *p =* .037).

**Figure 2:**
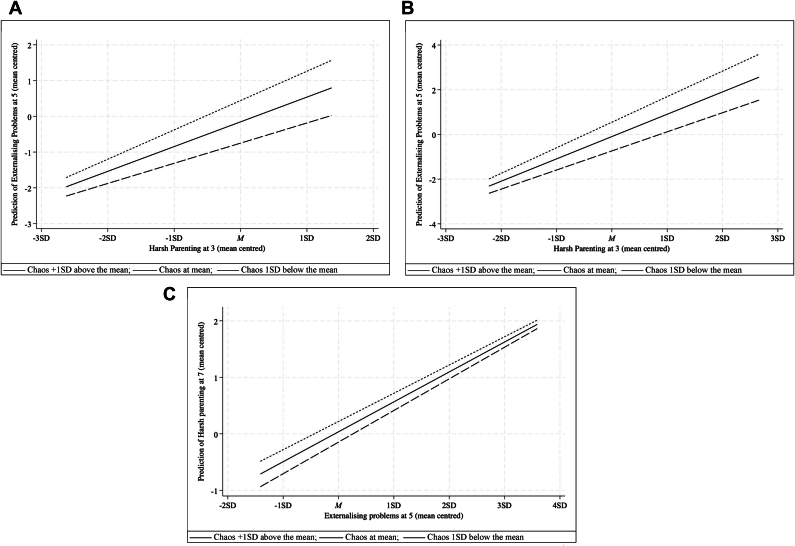
Moderation Effects (Marginal Effects) ***Note:****A) Harsh parenting at age 3 years on Externalizing Problems at age 5 years, moderated by Chaos at 3 years. B) Harsh Parenting at age 5 years on Externalizing Problems at age 7 years, moderated by Chaos at 5 years; Child Externalizing Problems at age 5 years on Harsh Parenting at age 7 years, moderated by Chaos at 5 years.*

To best understand and illustrate the nature of these interactions, we considered marginal effects (discussed in Data Analysis). These are visualized in Figure 2, showing associations between harsh parenting and externalizing problems when chaos was at the mean, 1 SD lower than the mean, and 1 SD higher than the mean. Parent-on-child and child-on-parent associations between harsh parenting and externalizing problems over time were evident at all levels of chaos. For harsh parenting at age 3 years predicting externalizing problems at age 5 (Figure 2A) and harsh parenting at age 5 predicting externalizing problems at age 7 (Figure 2B), higher levels of harsh parenting and perceived chaos were positively associated with higher levels of later externalizing problems. At both timepoints, the effect of harsh parenting on children’s later externalizing problems was implied to be slightly stronger at higher levels of chaos (or weaker at lower levels). For the moderated influence of externalizing problems at age 5 years on harsh parenting at age 7 (Figure 2C), higher levels of externalizing problems and of perceived chaos were associated with higher levels of harsh parenting. Moreover, at lower levels of chaos, the influence of externalizing problems on later harsh parenting was suggested to be slightly stronger, or, alternatively, at higher levels of chaos, the influence of externalizing problems on later harsh parenting was lessened.

## Discussion

Capitalizing on a large longitudinal sample (ie, the Millennium Cohort Study), we examined household chaos as a moderator of the bidirectional associations between harsh parenting and children’s externalizing behaviors from early to mid childhood (ages 3, 5, and 7 years). Replicating previous findings, harsh parenting, externalizing problems, and chaos were mutually related within and across time.^24^ In line with our expectations, chaos was found to moderate the paths between harsh parenting and children’s behavior. Specifically, chaos interacted with harsh parenting at the first 2 time points (ages 3 and 5 years) to predict children’s externalizing problems 2 years later. Furthermore, household chaos and children’s externalizing problems interacted to predict later harsh parenting, in an unexpected but logical direction.

Weak bidirectional associations between harsh parenting and externalizing behavior through childhood were evident, even though our conservative model also accounted for within-construct stability, household chaos at ages 3 and 5 years. as well as our interactions and confounding variables. These findings provide robust support for Bell’s early proposition of parent-on-child and child-on-parent effects,^4^ Patterson’s model of coercive cycles,^6^ and numerous subsequent theoretical and empirical works.^5^^,^^7^^,^^8^^,^^10^ The existence of negative bidirectional dynamics between parents and children outside of the clinical context and over time is of high importance, as the majority of these families will not receive support to help them break these cycles. As such, the potential for the continuation of these detrimental mutual dynamics is considerable, prolonging and embedding patterns of behavior likely to convey long-term risks of mental health and other difficulties for both parent and child.^6^ Albeit not a primary focus, it is of additional interest that bidirectionality was evident in the associations between all 3 of our constructs at the early ages (3-5 years), and that paths between chaos, externalizing problems, and harsh parenting over time in all directions were of similar magnitude. These findings align with prior cross-sectional and longitudinal research exploring associations between chaos, parenting, and children’s behavioral development^24^ as well as the contributing role of individuals in the family to chaos in the home.^39^

Because household chaos has a role in disrupting proximal family processes,^19^ our core hypotheses focused on the potential moderating role of chaos in parent-on-child and child-on-parent influences. We expected moderation of these bidirectional processes to be likely, because perceived chaos can reduce the efficiency and effectiveness of self-regulation for both parents and children.^21^ As expected in our community sample, we found weak interactions between chaos and concurrent harsh parenting in association with child externalizing problems 2 years later. These findings suggested that household chaos may serve to exacerbate the relationship between parental harshness and children’s later externalizing problems, mirroring the pattern of results commonly found in simpler analyses from community samples.^24^

We were also interested in chaos and externalizing problems as potential interacting processes in the prediction of later harsh parenting. Again, the reduced capacity for regulation in parents and children was relevant to this hypothesis,^21^ as were prior findings of socioeconomic disadvantage playing this role.^20^ We found small-magnitude interaction effects as expected but did not anticipate the direction of effects implied. We had assumed that chaos would have a similarly exacerbating effect on the role of externalizing problems in eliciting more harsh parenting over time as it did on the role of harsh parenting on children’s externalizing problems. Instead, these findings implied that in higher-chaos homes children’s externalizing problems had a weaker influence on harsh parenting, or, on the other hand, that externalizing problems had a greater influence on parental harshness in households in which parents reported a lower level of chaos. Our findings align with the only known previous study in this area,^15^ despite age range differences and that perceptions of chaos were measured at a single, end timepoint rather than the first 2 timepoints alongside parenting and child behavior as we have done here.

Several interpretations are possible. It has been argued^40^ that the influence of chaos on increasing child behavior problems is a function of children in chaotic homes filtering out “excessive” stimulation, be it detrimental or beneficial (eg, positive parenting) for their behavior. We speculate that this may be true of parents as well: that is, parents who perceive high chaos in their home may filter out the excessive stimulation of their children’s externalizing problems and thus may be less reactive to it. A related interpretation is that when parents perceive chaos to be high, their attribution of the child’s externalizing problems as the source of proximal stress may be less pronounced, as it may be more difficult to differentiate individual stressors. This may mean that these parents are less likely to be directly reactive to the child compared to those perceiving chaos as low, where parents may be more likely to attribute the proximal stress source to the child’s behavior and to parent the child more harshly. A third interpretation is that parents with lower levels of chaos at home may be those more prone to needing to control the organization, routines, and noise of their home, and who may be especially sensitive to their child’s externalizing behaviors, leading them to be more reactive and harsher as a result. This interpretation relates other explanations of similar findings^15^: that parents in low-chaos homes may be more keyed into externalizing problems and may parent more harshly in response. Of relevance to these interpretations is the aforementioned move away from deficit-model biases—whereby chaos and other contextual factors are seen as “risk” and necessarily detrimental to children’s outcomes—recognizing contextual sensitivity in individuals’ development that may be adaptive or maladaptive.^23^ Another point of note is that our expectation of the direction of effects was partly based on prior work showing socioeconomic disadvantage to exacerbate the association between children’s behavior problems and parenting.^20^ Our unexpected finding may simply be indicative of our inclusion of child-on-parent rather than just parent-on-child influences, bringing us closer to the complexity of real-world processes in the family, and moving us away from assumptions of deficit. We emphasize that our interpretations are speculative and warrant more research, particularly as our interactions were of small effect.

In considering the interaction between externalizing problems and chaos in association with later harsh parenting, findings were of similar magnitude at the earlier and later ages, although at age 5 to 7 years the interaction term reached significance. Because of the spurious nature of *p* value cut-off points, it is important that we do not overinterpret these findings, and we reiterate that replication of our research is crucial. However, if replication continues to suggest that the interactive effects do not occur in the preschool years but do occur later in childhood, we have a cautious, feasible interpretation. Specifically, it may be that, for families in which children’s behaviors influence the chaotic feel of the home environment,^39^ the home is perceived as relatively less chaotic during the hours that they are at school (from age 4 to 5 years in the United Kingdom) and relatively more chaotic when they are home. This may mean that the detrimental effects of children’s externalizing problems on parental harshness increases when they are at home, that is, with the increase to perceptions of chaos. These findings warrant future research to disentangle their implications.

Our study has many strengths, not least the use of a prospective longitudinal study with identical data at 3 timepoints over the preschool to middle-childhood years. This period is pertinent for understanding the mechanisms in which we were interested, because it is a time for expected changes in family dynamics as well as in children’s behavior. Moreover, the scale of the study provided us with statistical power to detect the hypothesized interactions of interest. Interactions between constructs are more difficult to detect in field/community samples than in experimental samples that have more control of variables and minimal complexity from real-world contexts.^41^ Failures to detect existing interactions (type II error) in these contexts are likely, in part because of their magnitude^42^; yet, with appropriate caution, interactions of small effect can be pertinent for theory and for practice.^36^

Despite the study’s strengths, we acknowledge some weaknesses. In particular, the nature of the study does not allow for detailed consideration of any of our constructs, which could reveal key associations over time. Although global assessments over many years cannot capture more nuanced micro-level dynamics between parents and children where we might expect to show stronger effect sizes, they capture crucial family systems seen to be predictive of long-term outcomes in community samples. We detected moderation effects despite the limitations of the time between measurement points, leading us to encourage future work exploring these research questions in different samples. Finally, we relied on parent report for all measures used in this secondary data analysis, likely confounded by shared-method variance. However, we argue that this weakness is also a strength, as parent perceptions are important in the interpretation of our results.

If replicated, our findings have key implications for understanding parent perceptions of household chaos in the general population, and perhaps suggest limiting aspects of chaos that are within parental control. Notably, however, there is a need to avoid preconceived notions of objectively “appropriate” levels of noise or bustle in the home, favoring attention to the home environment as it is subjectively perceived, with individual needs and responses reflected. In doing so, there may be potential to prevent exacerbation of harsh parenting effects on child behavior, improving individual and family prognoses, and ultimately reducing clinical burden. Moreover, our findings have implications for clinical work and practice where interrupting negative cycles between parents and children is already a common focus (eg, through parenting programs). We suggest that consideration of parent perceptions of the broader family environment with a contextual lens may add to the effectiveness of such intervention and improve health equity.

Our motivation to explore moderating factors of mutually influential dynamics between parents and children has its roots in better understanding risks for the continuation of these negative dynamics, with a view to understanding how to prevent downward spirals for parents and children inside and outside of the clinical context. By doing so, we are ambitious to facilitate not just an understanding of the interruption of negative cycles, but also factors that may reverse or, better still, prevent them. The mutuality of parent and child behaviors has enormous potential for generating positive cycles as well, whereby parents and children have mutual influence on positive, caring, and prosocial behaviors.

## CRediT authorship contribution statement

**Bonamy R. Oliver:** Writing – review & editing, Writing – original draft, Visualization, Project administration, Methodology, Investigation, Funding acquisition, Formal analysis, Data curation, Conceptualization. **Jon Heron:** Writing – review & editing, Methodology, Funding acquisition, Formal analysis. **Jasmine A.L. Raw:** Writing – review & editing. **Jane Gilmour:** Writing – review & editing, Funding acquisition. **Emily Midouhas:** Writing – review & editing, Writing – original draft, Visualization, Project administration, Investigation, Funding acquisition, Data curation, Conceptualization.
