# Supplementary material for: Changing Family Dynamics Through Childhood: Exploring Household Chaos as a Moderator of Bidirectional Effects Between Parent and Child Behaviors
Source: JAACAP Open. 2025 Aug 12;4(1):101–10. doi: 10.1016/j.jaacop.2025.08.001 (PMC12925893; doi:10.1016/j.jaacop.2025.08.001)
Supplement: Supplemental.Tables [file mmc1.docx]

**Supplementary Table S1:** *Details of Missing Data for Primary Variables*

To assess systematic missingness in our primary variables, we conducted logistic regressions predicting missingness from our confounders. Missingness was consistently higher among main-carers without a university degree or higher education; for all variables except chaos at age 5, missingness was associated with being below the poverty line; and for all variables except chaos at age 3, missingness was higher among main-carers identifying their ethnicity as non-white. Additionally, missingness in harsh parenting at age 7 was more likely for male children. Missingness for the primary variables is summarized below, with significant predictors at *p*<.05 shown by superscript indicators.

|  |  | |  | % Missing data |
| --- | --- | --- | --- | --- |
| Externalizing Problems | | at 3 years^a,b,d,e^ | | 14.73 |
|  | | at 5 years^a,b,d,e,f,h^ | | 14.41 |
|  | | at 7 years^a,b,d,e,f^ | | 21.63 |
| Harsh Parenting | | at 3 years^a,b,d,e,f,g,h^ | | 24.06 |
|  | | at 5 years^a,b,d,e,f^ | | 17.36 |
|  | | at 7 years^a,b,c,d,e,f,g,h^ | | 27.27 |
| Chaos | | at 3 years^a,b,h^ | | 9.75 |
|  | | at 5 years^a,d^ | | 11.70 |

*Note: ^a^main-carer educated at degree level or higher; ^b^poverty status; ^c^child female; ^d^white; ^e^mixed; ^f^Indian; ^g^Pakistani/Bangladeshi; ^h^Black; ^i^Other. All indicators are dummy coded as follows: main-carer education (1=university degree or higher by age 7), poverty status (1=below poverty line set for equivalized net family income at 60% of UK national median), child sex assigned at birth (1=female participant) and child ethnicity.*

**Supplementary Table S2.** *Path Model Results: Associations Between Externalising Problems and Harsh Parenting Over Time Moderated By Household Chaos*

For ease of reference, Table 2 (main manuscript) presents parameter estimates for core variables only, but these are based on the full model accounting for all confounders. Below, we present these same estimates as well as showing those for confounders.

|  |  | | Standardised Estimate | S.E. | *p* |
| --- | --- | --- | --- | --- | --- |
| Predicting Externalizing Problems at 5 years | | |  |  |  |
|  | Externalising problems at 3 years | | 0.55 | 0.01 | < .001 |
|  | Harsh parenting at 3 years | | 0.04 | 0.01 | < .001 |
|  | Household chaos at 3 years | | 0.05 | 0.01 | < .001 |
|  | Harsh parenting*Household chaos at 3 years | | 0.02 | 0.01 | .005 |
|  | Child assigned female at birth^†^ | | -0.08 | 0.01 | < .001 |
|  | Parent educated to university degree or higher^†^ | | -0.06 | 0.01 | < .001 |
|  | Below poverty line | | 0.07 | 0.01 | <.001 |
|  | Ethnicity | |  |  |  |
|  |  | Black | 0.03 | 0.01 | .710 |
|  |  | Indian | 0.00 | 0.01 | .668 |
|  |  | Mixed | 0.02 | 0.01 | .035 |
|  |  | Other | 0.01 | 0.01 | .301 |
|  |  | Pakistani or Bangladeshi | -0.02 | 0.01 | .136 |
| Predicting Harsh Parenting at 5 years | | |  |  |  |
|  | Harsh parenting at 3 years | | 0.54 | 0.01 | < .001 |
|  | Externalising problems at 3 years | | 0.07 | 0.01 | < .001 |
|  | Household chaos at 3 years | | 0.03 | 0.01 | .002 |
|  | Externalising problems*Household chaos at 3 years | | -0.01 | 0.01 | .290 |
|  | Child assigned female at birth^†^ | | -0.04 | 0.01 | < .001 |
|  | Parent educated to university degree or higher^†^ | | 0.01 | 0.01 | .033 |
|  | Below poverty line | | -0.04 | 0.01 | <.001 |
|  | Ethnicity | |  |  |  |
|  |  | Black | 0.01 | 0.01 | .410 |
|  |  | Indian | -0.01 | 0.01 | .216 |
|  |  | Mixed | 0.00 | 0.01 | .601 |
|  |  | Other | -0.01 | 0.01 | .493 |
|  |  | Pakistani or Bangladeshi | -0.02 | 0.01 | .241 |
| Predicting Chaos at 5 years | | |  |  |  |
|  | Household chaos at 3 years | | 0.41 | 0.01 | < .001 |
|  | Harsh parenting at 3 years | | 0.10 | 0.01 | < .001 |
|  | Externalising problems at 3 years | | 0.04 | 0.01 | < .001 |
|  | Child assigned female at birth^†^ | | -0.01 | 0.01 | .332 |
|  | Parent educated to university degree or higher^†^ | | -0.03 | 0.01 | < .001 |
|  | Below poverty line | | 0.09 | 0.01 | <.001 |
|  | Ethnicity | |  |  |  |
|  |  | Black | -0.01 | 0.01 | .286 |
|  |  | Indian | -0.02 | 0.01 | .082 |
|  |  | Mixed | -0.00 | 0.01 | .911 |
|  |  | Other | 0.00 | 0.01 | .938 |
|  |  | Pakistani or Bangladeshi | -0.06 | 0.01 | < .001 |
| Predicting Externalizing Problems at 7 years | | |  |  |  |
|  | Externalising problems at 5 years | | 0.66 | 0.01 | < .001 |
|  | Harsh parenting at 5 years | | 0.05 | 0.01 | < .001 |
|  | Household chaos at 5 years | | 0.03 | 0.01 | < .001 |
|  | Harsh parenting*Household chaos at 5 years | | 0.02 | 0.01 | .009 |
|  | Child assigned female at birth^†^ | | -0.07 | 0.01 | < .001 |
|  | Parent educated to university degree or higher^†^ | | -0.03 | 0.01 | < .001 |
|  | Below poverty line | | 0.05 | 0.01 | <.001 |
|  | Ethnicity | |  |  |  |
|  |  | Black | 0.03 | 0.01 | .001 |
|  |  | Indian | -0.01 | 0.01 | .364 |
|  |  | Mixed | -0.01 | 0.01 | .103 |
|  |  | Other | -0.01 | 0.01 | .492 |
|  |  | Pakistani or Bangladeshi | -0.02 | 0.01 | .082 |
| Predicting Harsh Parenting at 7 years | | |  |  |  |
|  | Harsh parenting at 5 years | | 0.59 | 0.01 | < .001 |
|  | Externalising problems at 5 years | | 0.10 | 0.01 | < .001 |
|  | Household chaos at 5 years | | 0.03 | 0.01 | < .001 |
|  | Externalising problems*Household chaos at 5 years | | -0.02 | 0.01 | .037 |
|  | Child assigned female at birth^†^ | | -0.03 | 0.01 | < .001 |
|  | Parent educated to university degree or higher^†^ | | 0.01 | 0.01 | .025 |
|  | Below poverty line | | -0.03 | 0.01 | .001 |
|  | Ethnicity | |  |  |  |
|  |  | Black | -0.02 | 0.01 | .134 |
|  |  | Indian | -0.01 | 0.01 | .320 |
|  |  | Mixed | 0.00 | 0.01 | .730 |
|  |  | Other | 0.00 | 0.01 | .955 |
|  |  | Pakistani or Bangladeshi | -0.02 | 0.01 | .138 |

*Note: Confounders were dummy-coded (see main manuscript)*
